# Kinetics of carnitine concentration after switching from oral administration to intravenous injection in hemodialysis patients

**DOI:** 10.1080/0886022X.2018.1455587

**Published:** 2018-04-04

**Authors:** Anna Suzuki, Yukinao Sakai, Kazumasa Hashimoto, Hirokazu Osawa, Shuichi Tsuruoka

**Affiliations:** a Department of Nephrology, Nippon Medical School Musashikosugi Hospital, Kawasaki, Japan;; b Department of Nephrology, Hakujikai Memorial Hospital, Tokyo, Japan;; c Department of Nephrology, Graduate School of Medicine, Nippon Medical School, Tokyo, Japan

**Keywords:** Carnitine, hemodialysis, bioavailability, pharmacokinetics, carnitine metabolism

## Abstract

Carnitine has high dialyzability and is often deficient in dialysis patients. This deficiency is treated by either intravenous (IV) or oral supplementation of carnitine. In this study, the mode of carnitine administration was changed from oral to IV in 17 hemodialysis (HD) patients, and the treatment was discontinued after 1 year. We found that the levels of total carnitine (TC), free-carnitine (FC), and acyl-carnitine (AC) significantly increased after 3 months of switching to IV administration (*p <* .05). After discontinuation of carnitine administration, the TC, FC, and AC levels decreased before dialysis. The average FC value was maintained at the normal levels until 9 months, but fell below the normal values when measured at the 12th month of discontinuation. In conclusion, carnitine was maintained at significantly high levels despite the smaller dose by IV infusion as compared with that by oral administration. We therefore suggest that our results be considered while determining both the carnitine administration route and the administration period in dialysis patients under clinical settings.

## Introduction

l-carnitine is a water-soluble amine (molecular weight 162) present in the mitochondria of the tissues of cardiac muscle, skeletal muscle, brain, and liver, among others, as free-carnitine (FC) or acyl-carnitine (AC) [1]. Carnitine has high dialyzability and is often deficient in dialysis patients, because these patients are undernourished due to inflammation. Energy may be produced by beta-oxidation or through the tricarboxylic acid (TCA) cycle by conveying long-chain fatty acids through the inner mitochondrial membrane to carnitine. In addition, carnitine bound to acyl-coenzyme A (CoA) inhibited ATP transfer and carbohydrate metabolism, returned them to CoA and AC, and assisted in energy production by transferring them to the outside of the mitochondria [2]. Carnitine is present as FC or AC in the blood. As CoA and free CoA ratio balance is preserved, the blood AC/FC ratio shows carnitine metabolism. Carnitine deficiency causes systemic disorders such as heart failure, anemia, and muscle symptoms. As carnitine is eliminated by hemodialysis (HD), dialysis patients are particularly susceptible to carnitine deficiency and, consequently, to hypotension [3] and erythropoietin low-responsiveness anemia [4,5].

In Japan, carnitine is used as a medicine for treating carnitine deficiency. This disorder is treated by either IV or oral supplementation of carnitine. In HD patients, particularly, IV administration is often provided after dialysis. However, there are no reports available on the appropriate carnitine doses, administration methods, and dosing period for dialysis patients. As oral administration of carnitine has low compliance and induces gastrointestinal symptoms, a need was recognized to switch to the IV administration mode. However, no study has conducted a detailed analysis of changes in plasma carnitine concentration after a change in the administration method or after discontinuation of carnitine administration in dialysis patients.

In this study, first, the mode of carnitine administration was changed from oral to IV in the same HD patient. Second, the treatment was discontinued 1 year after IV administration, after which the effective blood carnitine concentration in the patient was examined and compared with that at the beginning of dialysis.

## Materials and methods

### Patients

This was a single-center, one-way, open-labeled, prospective study. All patients provided informed consent to participate in the study after the study protocol and the associated risks were individually explained to each patient. The present study protocol was approved by the Ethical Committee of the Hakujikai Memorial Hospital (2015–002) and registered at the University Hospital Medical Information Network (UMIN No. 000025804). This study was designed in accordance with the Declaration of Helsinki.

#### Treatment

During this study, 17 stable-maintenance HD patients treated by oral dosing of l-carnitine [L-Cartin^®^ FF, Otsuka Pharmaceutical Co., Ltd., Tokyo, Japan; 200 mg, thrice a day (*n* = 5) or 300 mg, thrice a day (*n* = 12)] for at least 11 months were enrolled (12 men and 5 women; age: 61.6 ± 9.2; time on HD 8.3 ± 6.2 years). After obtaining written informed consent from the patient, they were switched to an IV dosing mode (L-Cartin^®^ FF, Otsuka Pharmaceutical Co., Ltd., Tokyo, Japan; 1000 mg/day, after every dialysis). The patients’ clinical information is given in Table 1. The mean blood flow rate during the HD session was 203.5 ± 26.7 mL/min for 3.68 ± 0.43 h. The IV dose was 1000 mg at the end of every dialysis.

**Table 1.** Patients’ baseline characteristics (*n* = 17).

Female (*n*)	5
DM (*n*)	5
Age (years)	61.6 ± 9.2
Weight (kg)	59.3 ± 9.9
HD duration (years)	8.3 ± 6.2
HD time (hours)	3.68 ± 0.43
Quantity of blood (ml/min)	203.5 ± 26.7
Dialyzer surface area (m^2^)	1.84 ± 0.27
KT/V	1.296 ± 0.1776
Systolic BP (mmHg)	137.5 ± 14.23
Diastolic BP (mmHg)	73.18 ± 11.83

Values are shown as the *n* or mean ± standard deviation.

DM: diabetes mellitus; HD: hemodialysis; BP: blood pressure.

### Measurement

Carnitine kinetics were evaluated by plasma sampling at 0 and 2 weeks, 3, 6 and 12 months of switching to the IV dosing and at 3, 6, 9 and 12 months after terminating the dosing (i.e., 15 and 24 months after the initiation of the study). Each sampling was performed at the beginning of the HD sessions. The carnitine kinetics were evaluated by determining the plasma concentrations of total carnitine (TC), AC, FC, and the AC/FC ratio, which evaluates the relative lack of FC.

The carnitine concentration was measured using the enzyme cycling method, and their normal levels for TC were 45–91 μmol/L, FC, 36–74 μmol/L, and AC, 6–23 μmol/L [6]. We did not change the dialysis conditions such as the dialysis time and session or the dialyzer membrane surface area.

### Statistical analyses

The measurement values are shown as mean ± standard deviation. The one-way analysis of variance (ANOVA) was performed on the longitudinal data to address its multiplicity. Dunnett’s multiple comparison test was used as the post-hoc test. *p* < .05 was considered statistically significant. All analyses were performed using the Prism Software (version 6; GraphPad Software, Inc., La Jolla, CA).

## Results

The study population of 17 patients, including 5 with diabetes, comprised 12 men and 5 women. All patients had anuria, 16 of which were prescribed with oral medicine in Angiotensin II Receptor Blocker (ARB) and one with statin. One patient with serious cardiovascular complication also showed unstable angina and underwent coronary artery bypass surgery for the same. The patient’s baseline characteristics are listed in Table 1, and the laboratory data are shown in Table 2. Except for changes in the concentration of sodium and chlorine, no significant change was noted during the observation period.

**Table 2.** Patients’ laboratory data.

Month	0	12	24	*p* value
BUN (mg/dL)	61.26 ± 14.92	59.48 ± 9.657	57.08 ± 12.67	.4088
Cr (mg/dL)	11.34 ± 3.115	11.15 ± 2.386	11.52 ± 2.760	.605
UA (mg/dL)	7.218 ± 0.8398	7.076 ± 0.7790	6.819 ± 1.044	.1934
Na (mEq/L)	139.8 ± 2.899	137.9 ± 3.071	139.6 ± 3.052	.0068
K (mEq/L)	4.824 ± 0.6486	4.847 ± 0.5490	4.750 ± 0.7465	.7738
Cl (mEq/L)	101.7 ± 2.910	100.6 ± 3.104	104.1 ± 2.670	.0004
Ca (mg/dL)	8.488 ± 0.4343	8.794 ± 0.6378	8.688 ± 0.5965	.0809
P (mg/dL)	4.929 ± 0.9538	5.047 ± 0.8171	4.719 ± 1.025	.4571
TP (g/dL)	6.371 ± 0.4469	6.594 ± 0.4465	6.331 ± 0.4542	.0872
Alb (g/dL)	3.688 ± 0.2522	3.841 ± 0.2526	3.725 ± 0.3941	.1003
T-Cho (mg/dL)	171.7 ± 23.56	175.9 ± 32.71	163.8 ± 35.65	.4089
HDL-C (mg/dL)	47.71 ± 13.62	48.06 ± 13.80	50.56 ± 14.98	.1638
LDL-C (mg/dL)	97.24 ± 16.06	100.3 ± 23.68	91.87 ± 27.62	.5115
TG (mg/dL)	137.0 ± 77.87	157.2 ± 119.2	100.7 ± 59.45	.0775
AST (U/L)	13.82 ± 5.582	12.88 ± 3.740	12.38 ± 3.948	.7145
ALT (U/L)	11.24 ± 6.713	9.294 ± 3.177	8.813 ± 4.167	.4496
ALP (U/L)	247.2 ± 41.34	222.6 ± 52.45	244.8 ± 80.63	.214
GTP (U/L)	16.94 ± 10.54	14.82 ± 6.002	14.19 ± 4.847	.1526
Fe (mg/dL)	68.35 ± 21.29	71.47 ± 18.82	62.75 ± 22.68	.4335
TIBC (mg/dL)	273.5 ± 40.82	265.5 ± 34.89	256.4 ± 36.89	.0872
Ferritin (ng/mL)	89.42 ± 75.04	104.5 ± 85.18	105.6 ± 117.7	.5269
WBC (/μL)	5872 ± 1873	5634 ± 1539	6082 ± 2043	.5053
RBC (10^4^/μL)	340.9 ± 19.82	350.0 ± 36.28	349.1 ± 41.38	.5566
Hb (g/dL)	10.70 ± 0.5454	10.94 ± 0.8565	10.83 ± 1.185	.6485
Ht (%)	32.42 ± 1.535	33.36 ± 2.344	32.88 ± 3.850	.5175
Plt (10^4^/μL)	22.49 ± 7.218	23.02 ± 6.700	20.88 ± 7.206	.1553
CRP (mg/dL)	0.1271 ± 0.1643	0.1824 ± 0.2947	0.4112 ± 0.8395	.2228

Values are shown as the *n* or mean ± standard deviation.

BUN: blood urea nitrogen; Cr: creatinine; UA: uric acid; TP: total protein; Alb: albumin; T-Cho: total cholesterol; HDL-C: high-density lipoprotein cholesterol; LDL-C: low-density lipoprotein cholesterol; TG: triglyceride; AST: aspartate aminotransferase; ALT: alanine aminotransferase; GTP: γ-glutamyl transpeptidase; TIBC: total iron binding capacity; CRP, c-reactive protein.

**Figure 1.**
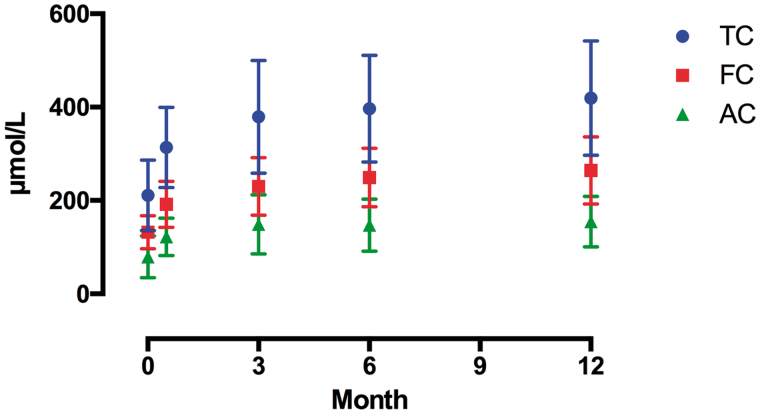
Levels of TC, FC, and AC after switching to the IV mode of administration were significant as per one-way ANOVA (*p* < .0001) and Dunnett’s multiple comparison tests (0 vs. 0.5, 0 vs. 3, 0 vs. 6, 0 vs. 12). TC: total carnitine; FC: free-carnitine; AC: acyl-carnitine.

**Figure 2.**
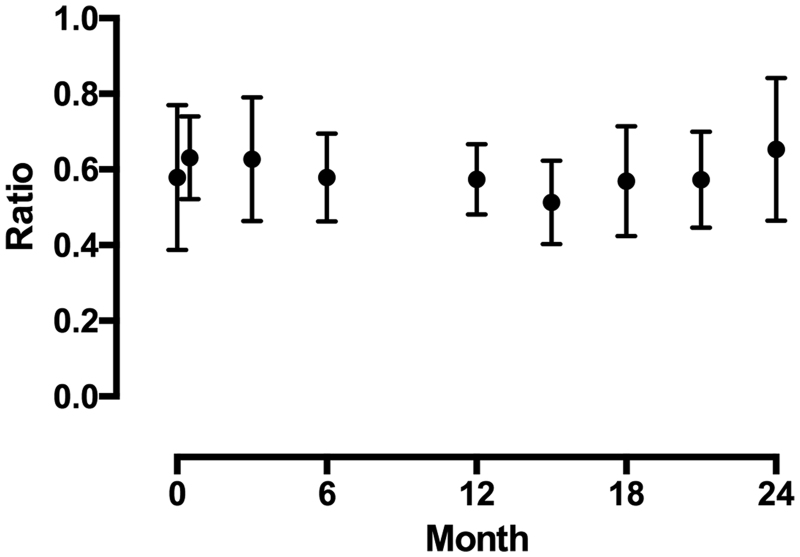
Change in the AC/FC ratio during and after discontinuation of carnitine administration. AC: acyl-carnitine; FC: free-carnitine.

**Figure 3.**
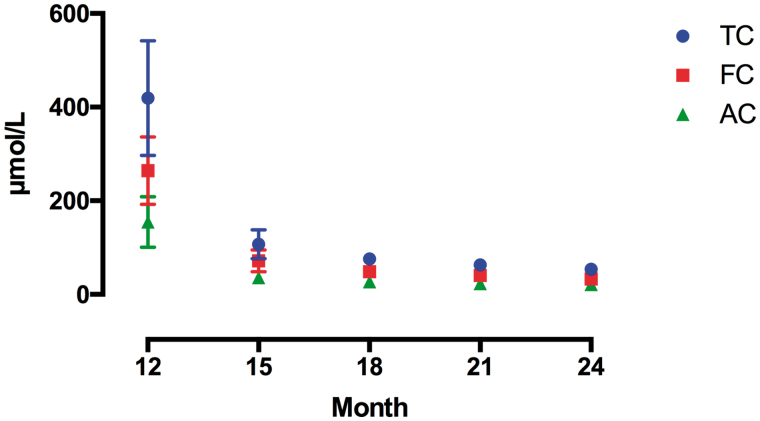
Levels of TC, FC, and AC after discontinuation of carnitine administration were found to be significant according based on the results of one-way ANOVA (*p* < .0001) and Dunnett’s multiple comparison tests (12 vs. 15, 12 vs. 18, 12 vs. 21, 12 vs. 24). TC: total carnitine; FC: free-carnitine; AC: acyl-carnitine.

The plasma concentrations of carnitine and its metabolites during the study period (24 months) are shown in Figures 1–3. Here, 0 month refers to the time when the patients were switched from oral to IV mode of administration.

The plasma concentrations of TC, FC, and AC at the beginning of the study were not statistically different among different doses, although it was higher in the group administered with 900 mg/day (TC 218.0 ± 75.76 μmol/L and 193.4 ± 90.0 μmol/L, FC 134.3 ± 31.1 μmol/L and 125.7 ± 48.1 μmol/L, AC 83.8 ± 46.9 μmol/L and 67.6 ± 42.4 μmol/L, 900 mg/day vs. 600 mg/day, respectively). The TC concentration was 210.8 ± 75.76 μmol/L before switching to IV administration (0 month) and 419.0 ± 123.0 μmol/L at the end of IV administration (12 months). The value at the 12th month (24 months) after discontinuation of administration was reduced to 53.80 ± 7.898 μmol/L.

The TC, FC, and AC levels significantly increased after 3 months upon switching to the IV mode of administration (TC, FC, AC: *p* < .0001) (Figure 1); however, there was no significant difference in the AC/FC ratio during the observation period (*p* = .1739) (Figure 2).

After discontinuation of carnitine administration before the dialysis, the TC, FC, and AC levels significantly decreased over 3 months, followed by slower decrease thereafter (*p* < .0001). The average FC value was maintained at the normal levels until 9 months, although the levels fell below the normal values when measured at the 12th month (Figure 3).

## Discussion

The AC, FC, and TC levels before the dialysis were above the normal levels with oral administration of carnitine. Maeda et al. [7] evaluated the carnitine concentrations after repeated oral doses before the patient underwent dialysis and found that dialysis reduced the plasma carnitine concentration, although high levels of carnitine were maintained after the dialysis. We obtained similar results in our study and hence confirmed that the oral mode of administration was effective.

Another report on the AC and FC levels before dialysis revealed higher than normal levels when carnitine was administered through IV infusion, which is in concordance with our results [8]. Fukami et al. [9,10] changed the mode of carnitine administration from oral to IV infusion and compared the blood carnitine levels in each mode. According to their reports, the plasma carnitine levels by IV dosing (500 mg/day, after every dialysis) was equal to that by oral dosing (900 mg/day, every day). When we increased the dosage, the plasma carnitine levels before and after dialysis increased, although the difference was not statistically different. Therefore, in this study, we changed the carnitine administration method in the same subjects under the same dialysis conditions for the first time and examined the plasma levels at different points in detail. After switching to an IV dosing mode, IV infusion mode resulted in well-maintained high plasma FC level as compared with that by oral administration, although the dosage was approximately half in IV infusion than in oral administration. This finding further ascertained that the estimated bioavailability of oral carnitine intake was only about 14%. Generally, the bioavailability of oral carnitine is prescribed by intestinal tract metabolism (the first-pass effect) [11]. In dialysis patients, absorption change due to intestinal edema, depressed metabolism in the tunica mucosa intestine tenuis, and urine toxin effect on the first-pass effect have been reported. Carnitine is a small molecule compound that is absorbed in the small intestine by diffusion and active transfer, and it is filtered once in the glomerulus of the kidney. However, >90% of the carnitine is reabsorbed in the renal tubule. Organic cation/carnitine transporter 2 (OCTN2), which is a cell membrane carrier, plays the main role in this absorption and reabsorption process [12]. While the bioavailability cannot be directly calculated from this result, this finding indicates an association between renal failure and carnitine first-pass effect. The AC levels increased after 2 weeks of switching to an IV dosing mode, but the levels were subsequently maintained at a steady state thereafter.

The AC/FC ratio is an index used to evaluate the carnitine metabolism. AC removal by dialysis is more difficult than FC removal, and patients with renal failure are believed to accumulate AC easily. As a result, even if we administered carnitine to improve the metabolism, the AC/FC level may not change appropriately. While it has been reported in the past that the AC/FC level significantly decreased 6 months after switching to the IV dosing mode [9,10], the decrease observed in our study did not reach statistical significance. This difference in results can be attributed to individual differences in the patient and in dialysis efficiency. The AC/FC level did not show any significant difference based on our hospital IV dosing period over 1 year, indicating that the rise in both FC and AC levels was approximately equivalent and that the carnitine metabolism may be promoted with an increase in the FC concentrations. In addition, the AC/FC ratio was not significantly different over 1 year after the discontinuation of carnitine administration.

After discontinuation of IV injection, FC was removed by dialysis. In this case, lower than normal values were only observed for the first time at 12 months after discontinuation. As carnitine is diffused into the extracellular space, 98% of carnitine is present in the muscle tissues and only 0.5% of it is present in the blood. Thus, carnitine metabolism appears to be promoted by 1 year of IV dosing, and time was required to supply the whole body with carnitine despite increased blood concentrations. It was difficult to ascertain whether the tissue supplementation was sufficient. In this study, the carnitine levels fell below the normal values after 1 year of discontinuation.

Takeuchi et al. [13] reported that the plasma carnitine concentration increased to several times the normal value at 12 weeks after carnitine administration, lasting up to 6 months [13]. Even in our study, the increase in the plasma concentration ceased after 3 months of IV injection, thereafter achieving a steady state. In addition, the plasma carnitine concentration decreased at 3 months after discontinuation of administration, but this reduction reached values lower than that in a healthy adult at only 12 months. It was not until the 12th month after discontinuation of administration that the level reached below the normal value, suggesting re-administration thereafter.

Supplementation of carnitine has been reported to facilitate decrease in hypotension during dialysis and improvement in the cardiac function, cardiomegaly, and muscle cramps [14–17]. In addition, carnitine was maintained at a stable level in the red blood cells. Moreover, l-carnitine administration has been reported to be effective in increasing the red blood cell lifespan through improvement of the erythrocyte membrane fat metabolism [3], improvement of erythrocyte cell membrane stabilization [18], and erythropoietin low-responsiveness anemia in renal diseases [13,19]. Furthermore, l-carnitine was found to be effective as an antioxidant and in inflammatory reaction suppression, which could improve the nutritional status [4,5,20–23]. In general, this effect may be evaluated by subjective symptoms; however, in this study, the subject’s symptoms had already disappeared at the start of this study.

## Limitation

Our study has some limitations. First, as this was a non-randomized study conducted at a single center, the sample size was too small to allow for robust statistical analyses. Second, this study did not consider the individual differences in the physique of the subject and the dialysis efficiency. Finally, because it is a medical treatment based on insurance treatment, the patient selection was biased.

## Conclusions

This study reported about HD patients in whom the mode of carnitine supplementation was switched from oral to IV. We discontinued the administration for 1 year and observed long-term changes in the plasma carnitine concentration before dialysis and its metabolite levels in detail. The IV infusion maintained the carnitine levels at significantly high concentrations despite the use of a smaller dose as compared with that in oral administration. Our results would help determine both the optimal carnitine administration route and the administration period in dialysis patients under clinical settings; however, there is a need to further study the same for measuring the optimal carnitine bioavailability.

## References

[1] Ozawa H. Nutrition management in severe motor and intellectual disabilities. J Jpn Soc Severe Motor Intellect Disabil. 2012;37:101–106.

[2] Stumpf DA, Parker WD Jr, Angelini C. Carnitine deficiency, organic acidemias, and Reye’s syndrome. Neurology. 1985;35:1041–1045. 3892364 10.1212/wnl.35.7.1041

[3] Hurot JM, Cucherat M, Haugh M, et al Effects of L-carnitine supplementation in maintenance hemodialysis patients: a systematic review. JASN. 2002;13: 708–714. 11856775 10.1681/ASN.V133708

[4] Vesela E, Racek J, Trefil L, et al Effect of L-carnitine supplementation in hemodialysis patients. Nephron. 2001;88:218–223. 11423752 10.1159/000045993

[5] Savica V, Santoro D, Mazzaglia G, et al L-carnitine infusions may suppress serum C-reactive protein and improve nutritional status in maintenance hemodialysis patients. J Renal Nutr. 2005;15:225–230. 10.1053/j.jrn.2004.10.002 15827896

[6] Tsushima K, Kase N. Dialysis therapy and carnitine. Methods for the determination of carnitine in human serum. Rinshotoseki. 2000;16:167–173.

[7] Maeda K, Shinzato T, Yoshida H, et al Effects of L-carnitime administration on metabolism of short-chain fatty acid and long-chain fatty acid during hemodialysis. J Jpn Soc Dial Ther. 1987;20:457–463.

[8] Kudoh Y, Aoyama S, Torii T, et al L-carnitine kinetics in chronic hemodialysis patients: comparison between oral and intravenous supplementation. J Biochem Pharmacol Res. 2014;2:117–124.

[9] Fukami K, Sakai K, Kaida Y, et al Effects of oral or intravenous L-carnitine administration on serum carnitine levels and clinical parameters in hemodialysis patients. J Jpn Soc Dial Ther. 2014;47:367–374.

[10] Fukami K, Yamagishi S, Sakai K, et al Effects of switching from oral administration to intravenous injection of L-carnitine on lipid metabolism in hemodialysis patients. Clin Kidney J. 2014;7:470–474. 25878778 10.1093/ckj/sfu082 PMC4379340

[11] Kolars JC, Watkins PB, Merion RM, et al First-pass metabolism of cyclosporin by the gut. Lancet. 1991;338:1488–1490. 1683920 10.1016/0140-6736(91)92302-i

[12] Kato Y, Sugiura M, Sugiura T, et al Organic cation/carnitine transporter OCTN2 (Slc22a5) is responsible for carnitine transport across apical membranes of small intestinal epithelial cells in mouse. Mol Pharmacol. 2006;70:829–837. 16754783 10.1124/mol.106.024158

[13] Takeuchi M, Kiyohara M, Machida H, et al Effects of levocarnitine in hemodialysis patients. J Jpn Soc Dial Ther. 2012;45:955–963.

[14] Higuchi T, Abe M, Yamazaki T, et al Levocarnitine improves cardiac function in hemodialysis patients with left ventricular hypertrophy: a randomized controlled trial. Am J Kidney Dis. 2016;67:260–270. 26508680 10.1053/j.ajkd.2015.09.010

[15] Sakurabayashi T, Miyazaki S, Yuasa Y, et al L-carnitine supplementation decreases the left ventricular mass in patients undergoing hemodialysis. Circ J. 2008;72:926–931. 18503218 10.1253/circj.72.926

[16] Kudoh Y, Aoyama S, Torii T, et al Hemodynamic stabilizing effects of L-carnitine in chronic hemodialysis patients. Cardiorenal Med. 2013;3:200–207. 24454315 10.1159/000355016 PMC3884187

[17] Ahmad S, Robertson HT, Golper TA, et al Multicenter trial of L-carnitine in maintenance hemodialysis patients. II. Clinical and biochemical effects. Kidney Int. 1990;38:912–918. 2266675 10.1038/ki.1990.290

[18] Wanic-Kossowska M, Kazmierski M, Pawliczak E, et al Combined therapy with L-carnitine and erythropoietin of anemia in chronic kidney failure patients undergoing hemodialysis. Polskie Archiwum Medycyny Wewnetrznej. 2007;117:14–19. 17642201

[19] Matsumoto Y, Amano I, Hirose S, et al Effects of L-carnitine supplementation on renal anemia in poor responders to erythropoietin. Blood Purif. 2001;19: 24–32. 11114574 10.1159/000014474

[20] Trovato GM, Iannetti E, Murgo AM, et al Body composition and long-term levo-carnitine supplementation. La Clinica Terapeutica. 1998;149:209–214. 9842104

[21] Duranay M, Akay H, Yilmaz FM, et al Effects of L-carnitine infusions on inflammatory and nutritional markers in haemodialysis patients. Nephrol Dial Transpl. 2006;21:3211–3214. 10.1093/ndt/gfl356 16861734

[22] Fatouros IG, Douroudos I, Panagoutsos S, et al Effects of L-carnitine on oxidative stress responses in patients with renal disease. Med Sci Sports Exerc. 2010;42:1809–1818. 20216464 10.1249/MSS.0b013e3181dbacab

[23] Tabibi H, Hakeshzadeh F, Hedayati M, et al Effects of l-carnitine supplement on serum amyloid A and vascular inflammation markers in hemodialysis patients: a randomized controlled trial. J Renal Nutr. 2011;21:485–491. 10.1053/j.jrn.2011.01.001 21439850

